# PepNN: a deep attention model for the identification of peptide binding sites

**DOI:** 10.1038/s42003-022-03445-2

**Published:** 2022-05-26

**Authors:** Osama Abdin, Satra Nim, Han Wen, Philip M. Kim

**Affiliations:** 1 Department of Molecular Genetics, University of Toronto, Toronto, ON M5S 3E1 Canada; 2 Donnelly Centre for Cellular and Biomolecular Research, University of Toronto, Toronto, ON M5S 3E1 Canada; 3 Department of Computer Science, University of Toronto, Toronto, ON M5S 3E1 Canada

**Keywords:** Machine learning, Molecular modelling

## Abstract

Protein-peptide interactions play a fundamental role in many cellular processes, but remain underexplored experimentally and difficult to model computationally. Here, we present PepNN-Struct and PepNN-Seq, structure and sequence-based approaches for the prediction of peptide binding sites on a protein. A main difficulty for the prediction of peptide-protein interactions is the flexibility of peptides and their tendency to undergo conformational changes upon binding. Motivated by this, we developed reciprocal attention to simultaneously update the encodings of peptide and protein residues while enforcing symmetry, allowing for information flow between the two inputs. PepNN integrates this module with modern graph neural network layers and a series of transfer learning steps are used during training to compensate for the scarcity of peptide-protein complex information. We show that PepNN-Struct achieves consistently high performance across different benchmark datasets. We also show that PepNN makes reasonable peptide-agnostic predictions, allowing for the identification of novel peptide binding proteins.

## Abstract (web summary)

PepNN integrates an attention-based deep learning module with transfer learning to predict the peptide binding sites of input proteins and the binding ability of proteins lacking a known peptide ligand.

## Introduction

Interactions between proteins and peptides are critical for a variety of biological processes. A large fraction of protein–protein interactions are mediated by the binding of intracellular peptide recognition modules (PRMs) to linear segments in other proteins^1^. Moreover, peptide ligands binding to extracellular receptors have important functions^2^. In total, it is estimated that there are roughly 10^4^ human proteins that contain at least one PRM^3^ and that there are over 10^6^ peptide motifs encoded in the human proteome^1^. Disruption of these interactions and their regulation can consequently result in disease; for instance, many proteins with PRMs harbour oncogenic mutations^4^. It has also been shown that viral proteins encode peptidic motifs that can potentially be used to hijack host machinery during infection^5^.

In the absence of ample experimental data including solved structures, gaining molecular insight into these interactions and their associated disease states is contingent on the ability to model peptide binding computationally. This has been a difficult problem that has traditionally been approached with peptide-protein docking^6^. One widely used peptide docking tool is FlexPepDock, a Rosetta protocol that refines coarse-grain peptide-protein conformations by sampling from the degrees of freedom within a peptide^7^. In general, benchmarking studies have shown that peptide docking approaches often fail to accurately identify the native complex conformation^8–10^, indicating that this problem remains unsolved; current approaches are limited by the high flexibility of peptides as well the inherent error of scoring heuristics^6^. Machine learning approaches provide potential alternatives to docking, as they can sidestep the issue of explicit enumeration of conformational space and can learn scoring metrics directly from the data.

A number of machine learning approaches have been applied to the problem of predicting the binding sites of peptides with varying amounts of success, including random forests and support vector machines (SVMs)^11–16^. More recently, a deep convolutional neural network was developed that takes as input a voxelized protein structure and outputs predicted binding sites on the protein, which achieved high accuracy^17^.

Here, we present an alternative deep learning architecture that allows for both sequence and structure-based prediction of peptide binding sites and performs the latter in a rotationally and translationally invariant manner. In particular, we develop an architecture that is partially inspired by the Transformer, a model that primarily consists of repeated multi-head attention modules^18^. These modules are effective at learning long-range dependencies in sequence inputs and have been successfully adapted to graph inputs^19^. Graph neural networks in general have had success on various related problems, including protein design^19,20^. Importantly, we build upon attention to develop reciprocal attention, a variant that updates two input encodings based on dependencies between the two encodings, while maintaining symmetry in the updates. This architecture updates the embedding of both residues participating in an interaction simultaneously, and reflects the fact that the conformation of a bound peptide depends on the interacting protein target^21^.

One significant hurdle to the development of deep learning approaches for the modelling of peptide-protein complexes has been the paucity of available training data. To overcome this problem, we exploit available protein–protein complex information, thereby adding an order of magnitude more training data. The “hot segment” paradigm of protein-protein interaction suggests that the interaction between two proteins can be mediated by a linear segment in one protein that contributes to the majority of the interface energy^22^. Complexes of protein fragments with receptors thus represent a natural source of data for model pre-training. In addition, the idea of pre-training contextualized language models has recently been adapted to protein biology for the purpose of generating meaningful representations of protein sequences^23,24^. The success of these approaches provides an opportunity to develop a strictly sequence-based peptide binding site predictor.

In this study, we integrate the use of contextualized-language models, available protein–protein complex data, and a task-specific attention-based architecture, to develop parallel models for both structure and sequence-based peptide binding site prediction: PepNN-Struct and PepNN-Seq. Comparison to existing approaches reveals that our models perform better in most cases. We also show that the developed models can make reasonable peptide-agnostic predictions, allowing for their use for the identification of novel peptide binding sites.

## Results

### Parallel models for structure and sequence-based peptide binding site prediction

**Fig. 1:**
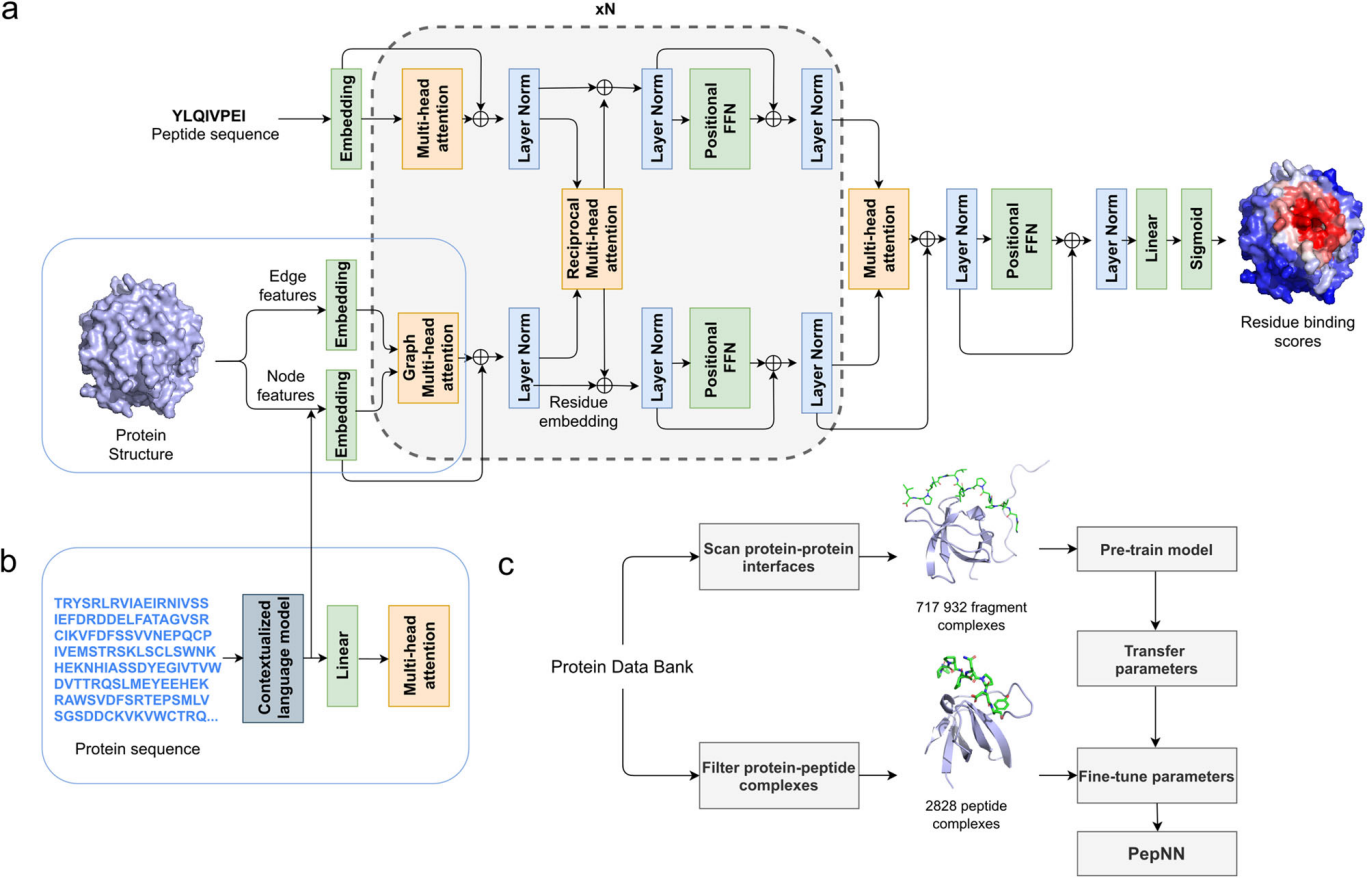
Model architecture and training procedure. **a** Attention layers are indicated with orange; normalization layers are indicated with blue and simple transformation layers are indicated with green. **b** Input layers for PepNN-Seq. **c** Transfer learning pipeline used for model training.

PepNN takes as input a representation of a protein as well as a peptide sequence, and outputs residue-wise scores representing the confidence that a particular residue is part of a peptide-binding site (Fig. 1a, b). The PepNN-Seq and PepNN-Struct architectures are based in part on the Transformer and a graph variant of the Transformer^18,19^. PepNN-Struct makes use of graph attention layers to learn a contextual representation of an input protein structure (Fig. 1a). PepNN-Seq generates predictions based solely on the input protein and peptide sequences (Fig. 1b).

PepNN differs from conventional Transformers in that it does not follow an encoder-decoder architecture. Encoding the peptide sequence independently from the protein representation would implicitly assume that all information about how the peptide binds the protein is contained within its sequence and this assumption is not concordant with the fact that many disordered regions undergo conformational changes upon protein binding^21^. In other words, a peptide’s sequence is insufficient by itself to determine its bound conformation. In fact, the same peptide can adopt different conformations when bound to different partners. Based on this, we introduce multi-head reciprocal attention layers, an attention-based module that simultaneously updates the peptide and protein embeddings while ensuring that the unnormalized attention values from protein to peptide residues are equal to the unnormalized attention values in the other direction. This ensures that the protein residues involved in binding have influence on the peptide residues and vice versa.

### Transfer learning results in large improvements in model performance

We used transfer learning in two ways to improve model performance. The first was to pretrain the model on a large protein fragment-protein complex dataset before fine-tuning it with a smaller dataset of peptide-protein complexes (Fig. 1c). To generate the fragment dataset, we scanned all protein–protein complex interfaces in the protein databank (PDB) that were deposited before 2018/04/30 using the Peptiderive Rosetta protocol^25^ to identify protein fragments of length 5-25 amino acids that contribute to a large portion of the complex interface energy (Supplementary Fig. 1). These fragment-protein complexes were filtered based on their estimated interface energy as well as the buried surface area to ensure that they had binding properties that were reasonably close to that of peptide-protein complexes. The second application of transfer learning was the use of a pre-trained contextualized language model, ProtBert^23^, to embed protein sequences. These high dimensional, information-rich, embeddings were used as input to PepNN-Seq and as part of the node encodings for PepNN-Struct (Fig. 1b).

**Fig. 2:**
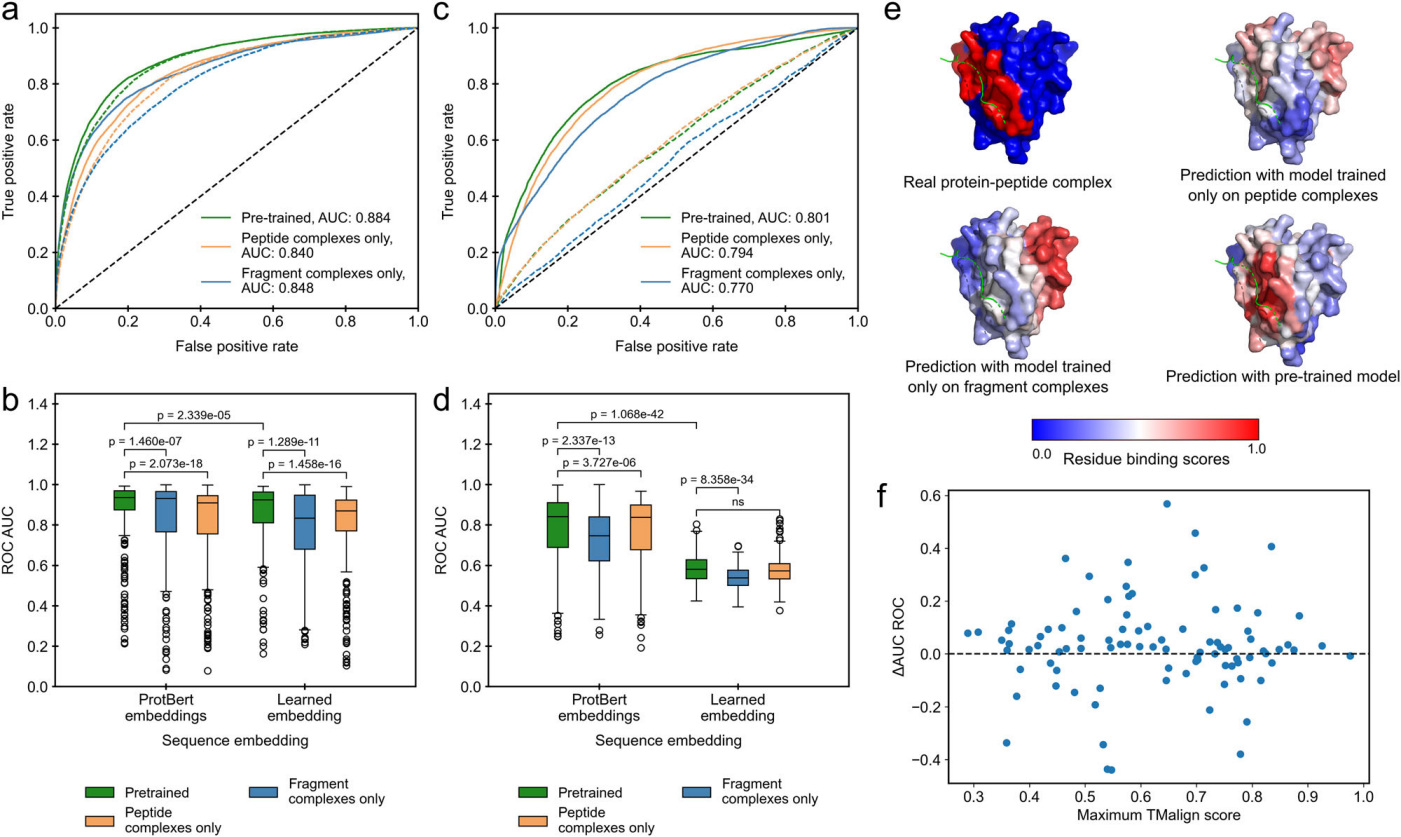
Impact of transfer learning on model performance on the peptide complex validation dataset. **a** ROC curves on all residues in the dataset using predictions from PepNN-Struct trained on different datasets with different sequence embeddings. Solid lines indicate models that use ProtBert embeddings. **b** Comparison of the distribution of ROC AUCs on different input proteins using predictions from PepNN-Struct trained on different datasets with different sequence embeddings (Wilcoxon signed-rank test, *n* = 311 protein-peptide complex structures). **c** ROC curves on all residues in the dataset using predictions from PepNN-Seq trained on different datasets with different sequence embeddings. Solid lines indicate models that use ProtBert embeddings. **d** Comparison of the distribution of ROC AUCs on different input proteins using predictions from PepNN-Seq trained on different datasets (Wilcoxon signed-rank test, *n* = 311 protein-peptide complex structures). **e** Predictions of the binding site of the SPOC domain of PHF3 (PDB code 6IC9) using PepNN-Struct trained on different datasets. **f** Relationship between the change in AUC ROC when PepNN-Struct is pretrained and the maximum TMalign score of chains in the test dataset with chains in the pre-training dataset. Boxplot centerlines show medians, box limits show upper and lower quartiles, whiskers are 1.5 the interquartile range and points show outliers.

To evaluate the impact of transfer learning on model performance, we trained PepNN-Struct and PepNN-Seq using different datasets, with and without providing the models with ProtBert embeddings. Pre-training PepNN-Struct resulted in a large improvement over models trained on only the fragment or peptide complex dataset, both in terms of overall binding residue prediction, and in terms of prediction for individual proteins (Fig. 2a, b). Model predictions on the SPOC domain of PHF3 demonstrate this difference in performance, as only the pre-trained variant of the model correctly predicts the peptide binding site (Fig. 2e). More generally, both the improvements in performance and absolute performance are not correlated to the structural similarity of the test set examples to proteins in the pre-training dataset (Fig. 2f, Supplementary Fig. 2). This illustrates that the pre-training step helps bring the parameters closer to an optimum for general peptide binding site prediction, rather than improving performance solely on examples that match patterns seen in the fragment-complex dataset.

Embedding protein sequences with ProtBert resulted in large performance improvements over learned embedding parameters for both PepNN-Struct and PepNN-Seq (Fig. 2a, d). Interestingly, pre-training on the fragment complexes did not have as large and impact on PepNN-Seq performance as it did on PepNN-Struct (Fig. 2a, d), indicating that PepNN-Struct likely learns from structural features in the pre-training dataset that are not captured in the ProtBert embeddings.

### PepNN outperforms an equivalent Graph Transformer

**Fig. 3:**
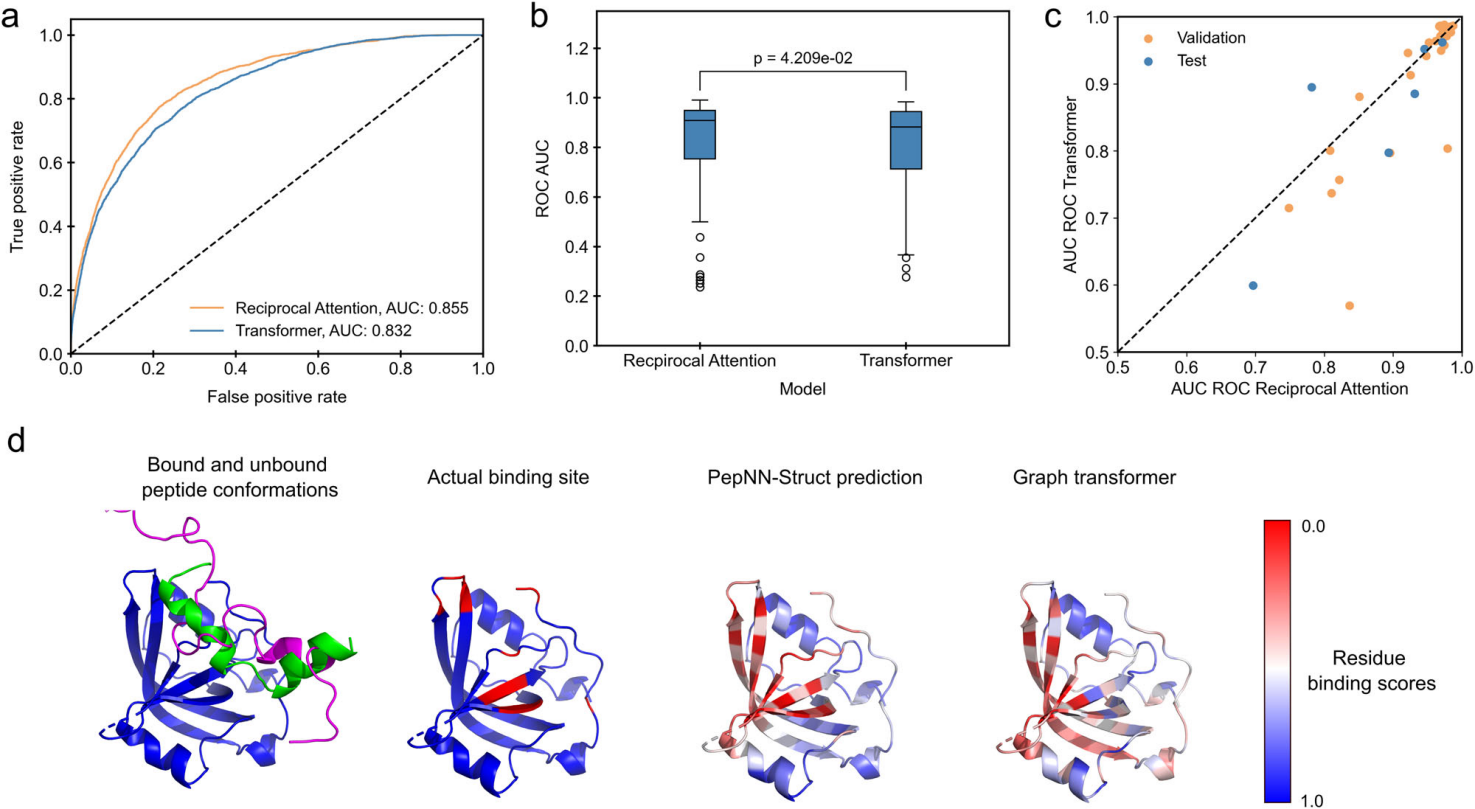
Comparison of PepNN-Struct and a Graph Transformer. **a** ROC curves on all residues in the TS092 dataset. **b** Comparison of distribution of ROC AUCs on different input proteins (Wilcoxon signed-rank test, *n* = 92 protein-peptide complexes). **c** Comparison of model performance on examples where bound peptides undergo conformational changes. **d** Prediction of the binding site of a P53 N-terminal peptide to RPA70N (PDB Code 2B3G). Unbound peptide conformation is shown in magenta (PDB Code 2LY4). Boxplot centerlines show medians, box limits show upper and lower quartiles, whiskers are 1.5 the interquartile range and points show outliers.

To evaluate the impact of reciprocal attention on model performance, we compared the performance of PepNN-Struct to a graph Transformer with the same hyperparameters, using different training procedures and input encodings (tuned by random search). We found that PepNN-Struct consistently achieves higher performance than the Graph Transform, irrespective of whether pre-training is done or ProtBert embeddings are included (Fig. 3a, b, Supplementary Fig. 3). As reciprocal attention can account for conformational changes of the peptide upon binding, we sought to investigate whether this effect is partly responsible for the performance of PepNN-Struct (see Methods). PepNN-Struct outperforms the Graph Transformer in almost all cases where a peptide has a Cα-RMSD of at least 2.5 Å from another structure (Fig. 3c). One such example is a peptide at the N-terminus of P53. This peptide undergoes a clear disorder-to-order transition upon binding (Fig. 3d). Compared to PepNN-Struct, the Graph Transformer predicts several residues that are not part of the peptide-binding sites (Fig. 3c).

### PepNN reliably predicts peptide binding sites

We evaluated the developed models on an independent test set, TS092, of recent peptide-protein complexes that are not redundant with the training and pre-training datasets, as well as three benchmark datasets from the previous studies^11,12,26^. For an unbiased estimate of model performance, PepNN was re-trained on the training datasets used in different studies prior to the evaluation of their test sets. Unsurprisingly, we found that PepNN-Struct consistently outperforms PepNN-Seq (Table 1). We do find, however, that both methods place the ground truth binding site in their top 10 predicted residues in most cases (Supplementary Table 1).

**Table 1:** Comparison of the developed model to existing approaches.

Test dataset	Training dataset size	Model	ROC AUC	MCC
TS092	2517	PepNN-Struct	**0.855**	0.409
		PepNN-Struct on AlphaFold models	0.850	0.409
		PepNN-Seq	0.781	0.272
	–	AlphaFold-Multimer	–	**0.605**
	–	AlphaFold-Gap	–	0.518
	475	PBRpredict-flexible^16^	0.587	0.084
		PBRpredict-moderate^16^	0.569	0.071
		PBRpredict-strict^16^	0.543	0.049
TS251	251	PepNN-Struct	**0.833**	0.366
		PepNN-Seq	0.769	0.277
		Interpep^11^	0.793	–
	–	AlphaFold-Multimer	–	**0.566**
	–	AlphaFold-Gap	–	0.470
TS639	640	PepNN-Struct	**0.868**	0.352
		PepNN-Seq	0.795	0.246
		PepBind^12^	0.767	0.348
	–	AlphaFold-Multimer	–	**0.450**
	–	AlphaFold-Gap	–	0.432
TS125	956	PepNN-Struct	**0.885**	0.390
		PepNN-Seq	0.794	0.259
		BiteNet_pp_^17^	0.882	0.435
	640	PepBind^12^	0.793	0.372
	1156	SPRINT-Str^26^	0.780	0.290
	1199	SPRINT-Seq^13^	0.680	0.200
	1004	Visual^15^	0.730	0.170
	–	AlphaFold-Multimer	–	**0.576**
	–	AlphaFold-Gap	–	0.448

Bolded values indicate the highest metric for each dataset.

To evaluate the performance of PepNN-Struct in the absence of co-crystallized protein structures, we additionally benchmarked its performance using AlphaFold models of receptors in TS092^27^. Performance is very similar when the solved protein structure is used and when the AlphaFold structural model is used (Table 1). In fact, in most cases the structure-based prediction using the AlphaFold model is still closer to the ground truth than the sequence-based prediction (Supplementary Fig. 4a). To assess whether this trend depends on the quality of the model generated by AlphaFold, the distributions of the model pLDDTs, a confidence score generated by AlphaFold, was compared for cases where PepNN-Struct performs better and cases where PepNN-Struct performs better. While the pLDDT is on average lower in cases where PepNN-Seq performs better (Supplementary Fig. 4b), the difference is not statistically significant (likely owing to the fact that AlphaFold generated consistently high-quality protein models on this test dataset).We additionally ran the sequence-based PBRpredict-Suite model on TS092^16^. All three variants of this model performed worse than PepNN on this dataset (Table 1) and notably, the observed performance was drastically lower than the performance reported in the original publication. This could potentially be due to the fact a smoothing approach was used to annotate binding sites in the PBRpredict-Suite study^16^, while binding site residues annotations were made based only on distance to peptide residues in this study. Most other designated peptide binding site prediction approaches lack programmatic access, and a portion rely on alignments to reference datasets that overlap with the test set. We hence used values reported in the literature for comparison. PepNN-Struct outperforms most peptide binding site prediction approaches, and performs comparably to the BitNet_pp_ approach, achieving a higher area under the receiver operator characteristic curve (ROC AUC), but performing worse in terms of Matthews correlation coefficient (MCC).

Despite not being trained on protein-peptide complexes, it has been additionally shown that AlphaFold, which has achieved ground-breaking success on the problem of protein structure prediction, can be adapted for protein-peptide complex modelling^27–29^. More recently, AlphaFold-Multimer was developed to model protein-protein complexes, and outperforms the initial AlphaFold implementation on this task^30^. AlphaFold-Multimer was additionally shown to outperform conventional docking approaches on the peptide-protein complex prediction^31^. Given that AlphaFold-based methods represent the current state-of-the-art in peptide-protein complex predictions, we compared binding site predictions generated by PepNN-Struct to binding sites derived from AlphaFold, (using the previously described gap method^32^) and AlphaFold-Multimer predictions. Consistent with what was shown in previous studies, AlphaFold and, to a greater extent, AlphaFold-Mulimer achieve remarkable accuracy in peptide-protein complex modelling in a large number of cases, achieving a higher MCC than PepNN overall (Table 1, Supplementary Figs. 5–6). On individual data points, there are many cases where the complex output by AlphaFold-Gap is completely detached from the receptor, however, and PepNN provides accurate predictions in many of these cases; in fact, AlphaFold-Gap much more frequently leads to poor predictions than PepNN (in 41% and 60% of cases in TS092 and TS125, respectively Alphafold provides predictions with MCC < 0.3, whereas PepNN only does so in 29% and 35%, respectively, see Supplementary Fig. 5). AlphaFold-Multimer, on the other hand, generates poor predictions less frequently, with only 18% and 24% of predictions having an MCC < 0.3 (Supplementary Fig. 6).

### Peptide-agnostic prediction allows the identification of putative novel peptide-binding proteins

**Fig. 4:**
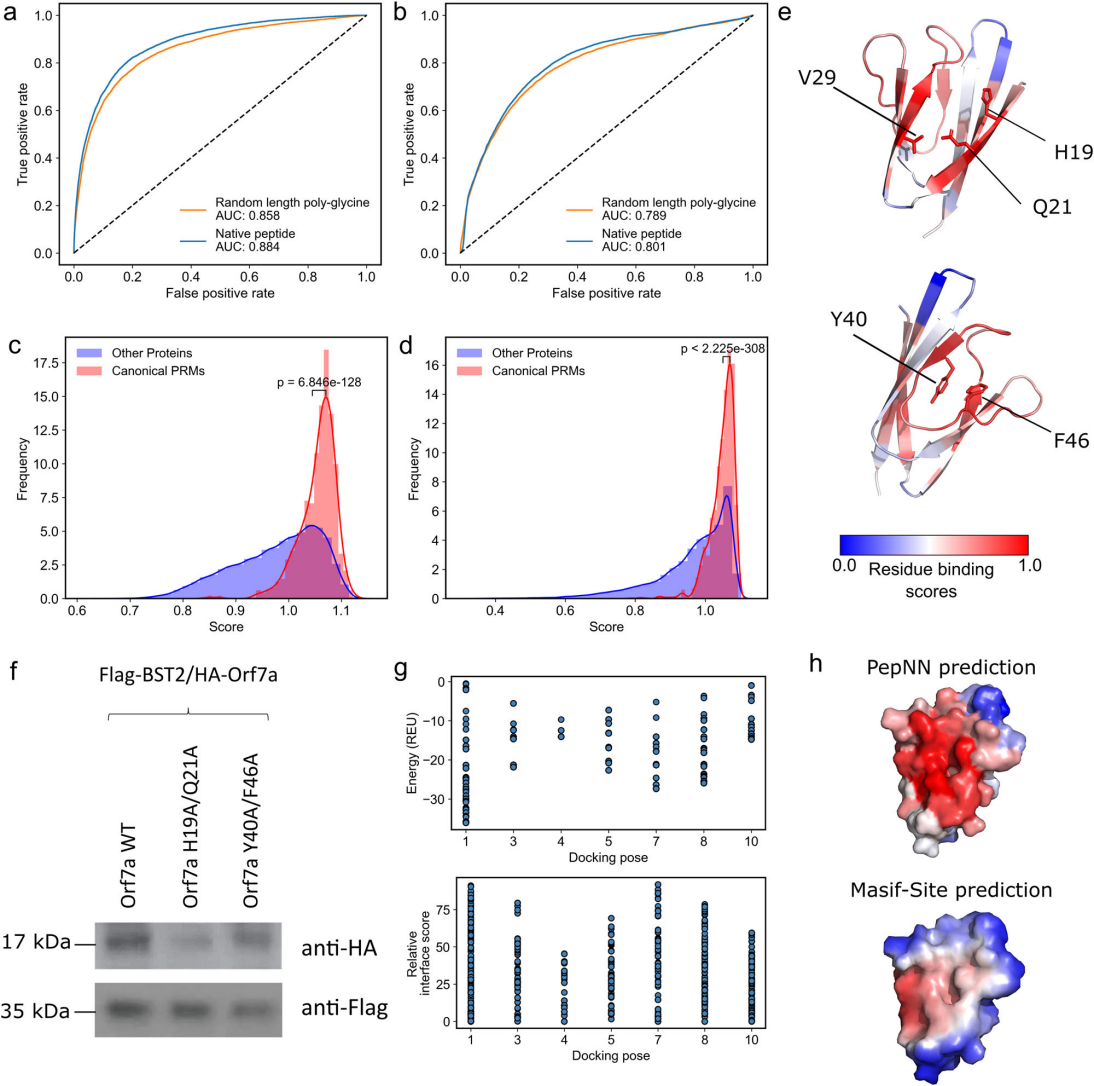
Peptide agnostic prediction using PepNN. **a** ROC curves on the validation dataset using PepNN-Struct with different input peptide sequences. **b** ROC curves on the validation dataset using PepNN-Seq with different input peptide sequences. **c** Scores assigned by PepNN-Struct to different domains in the PDB (Wilcoxon rank-sum test, 56,756 total protein chains). **d** Scores assigned by the PepNN-Seq to different domains in the reference human proteome (Wilcoxon rank-sum test, 92,141 total proteins). **e** ORF7a peptide binding site prediction and key residues at the predicted binding site and an alternate binding site. **f** Co-immunoprecipitation of wild type and mutant ORF7A with BST-2. **g** Energies and relative energy contributions of different fragments calculated using Peptiderive on ORF7a/BST-2 docking poses. **h** Binding site prediction on ORF7a using PepNN and BST-2.

To quantify the extent to which the model relies on information from the protein when making predictions, we tested the ability of PepNN-Struct and PepNN-Seq to predict peptide binding sites using random length poly-glycine peptides as input sequences. While the models did perform better when given the native peptide sequence than with a poly-glycine sequence (*p*-value < 2.2e−16 for both PepNN-Struct and PepNN-Seq, DeLong test), there was only a small overall decrease in the ROC AUC when a poly-glycine peptide was given (Fig. 4a, b). Comparing the probabilities that the model assigns to different residues shows that in both the case of PepNN-Struct, providing the native peptide increases the model’s confidence when predicting binding residues (Supplementary Fig. 7). Providing the native peptide sequence is thus important for reducing false negatives. Overall, these results suggest that while providing a known peptide can increase model accuracy, the model can make reasonable peptide-agnostic predictions and could potentially be used to identify novel peptide binders. To assess whether performing similar predictions is possible using AlphaFold-Multimer, predictions were generated using poly-glycine inputs, as done with PepNN. PepNN largely outperforms AlphaFold-Multimer when predictions are generated this way (Supplementary Fig. 8, overall MCCs 0.380 and 0.29 for PepNN and AlphaFold-Multimer respectively), likely owing to the fact that PepNN was trained specifically on peptide-protein complexes.

To quantify the model’s confidence that a protein is a peptide-binding module, we generated a score that takes into account the binding probabilities that the model assigns the residues in the protein, as well as the percentage of residues that the model predicts are binding residues with high confidence. To compute this score, a Gaussian distribution was fit to the distribution of binding residue percentages in each protein from the training dataset (Supplementary Fig. 9a). The resulting score was the weighted average of two metrics. The first is the average the top *n* residue probabilities and the second was the likelihood that a binding site would be composed of *n* residues based on the aforementioned distribution. For each protein, *n* was chosen to maximize this score. As done in a previous study^11^, the weight assigned to each component of the score was chosen to maximize the correlation between the MCC of the prediction for each protein in the validation dataset, and its score (Supplementary Fig. 9b, c). This was motivated by the fact that the confidence of the model should correlate with its correctness.

We used the models to predict binding sites for domains in every unique chain in the PDB not within 30% homology of a sequence in the training dataset or the pre-training set and domains in every sequence in the reference human proteome from UniProt^33^, not within 30% homology of a sequence in the training dataset or pre-training set. Domains were extracted by assigning PFAM^34^ annotations using InterProScan^35^ (Supplementary Data 1–2). To assess the capacity of the models to discriminate between peptide-binding modules and other domains, we compared the distribution of scores for canonical PRMs to that of other proteins. Previously defined modular protein domains^36^, and peptide binding domains^3^ were considered canonical PRMs. In both the case of the PDB and the human proteome, the scores for canonical PRMs were on average higher than other domains (Fig. 4c, d).

In total, PepNN-Struct assigns 31,868 domains in the PDB a score higher than the mean PRM score and PepNN-Seq assigns 20,442 domains in the human proteome a score higher than the mean PRM score. Analysis of the distribution of scores for different domains reveals that many DNA binding domains were assigned low scores on average by PepNN (Supplementary Data 3–4). This indicates that PepNN has the capacity to discriminate between different types of binding sites. There are, nonetheless, some nucleic acid binding domains with high scores (Supplementary Data 3–4) suggesting that there are false positives and that downstream computational and experimental work is required to validate putative peptide-binding sites.

One domain identified by PepNN-Struct is the sterile alpha motif (SAM) domain of the Deleted-in-liver cancer 1 (DLC1) protein (Supplementary Data 1). This domain was recently shown to be a peptide-binding module^37^, demonstrating the capacity of the model to identify novel peptide binders. Another interesting hit identified using PepNN-Struct is the ORF7a accessory protein from the SARS-Cov-2 virus (Supplementary Data 1). The model assigns the highest residue binding probabilities to residues between two beta-sheets at the N-terminal end of the protein (Fig. 4e). Validating this peptide binding site involves identifying a binding peptide and showing that the residues that comprise the binding site are necessary for the interaction. The ORF7a homolog from SARS-Cov has been shown to bind the ectodomain of the human BST-2 protein^38^. BST-2 binds and tethers viral particles to the cell membrane, thereby preventing viral exit^38^. It was shown that by binding BST-2, ORF7a prevents its glycosylation and thus reduces its ability to inhibit viral exit^38^. Given the fact that BST-2 forms a coiled-coil structure, it is possible that a linear segment along one of its helices binds to ORF7a at the predicted peptide-binding pocket.

As an unbiased test of this prediction, we performed global docking of BST-2 onto ORF7a using the ClusPro webserver^39,40^. In seven of the top ten poses, BST-2 was found to interact with ORF7a at the predicted binding site (Supplementary Fig. 10). Based on the predicted residue binding probabilities and the docking poses, alanine substitutions were introduced at two residues in the predicted binding site and two residues at an alternative binding site that was predicted with lower confidence (Fig. 4e). A co-immunoprecipitation assay demonstrated that the residues at the predicted binding site (H19A/Q21A) are necessary for ORF7a/BST-2 binding, corroborating the PepNN prediction (Fig. 4f). To assess whether this binding is mediated by a peptide, Peptiderive was run on the models generated by docking, where BST-2 binds at the predicted site. In most cases, the majority of the estimated interface energy can be attributed to a linear fragment, suggesting a peptide-mediated interaction (Fig. 4g). As additional validation, Masif-Site^41^, a geometric deep learning approach training to predict protein binding sites, was used to predict binding regions on the surface of ORF7a. Unlike PepNN, Masif-Site does not predict the N-terminal binding site (Fig. 4h), suggesting that it may be more characteristic of peptide binding sites than globular protein binding sites.

### Prediction of multiple peptide binding sites using PepNN

Many proteins interact with multiple peptides at different sites. Two such cases were found in TS092, where a single protein binds two peptides simultaneously (Supplementary Fig. 11a, b). When PepNN-Struct is used to predict binding sites in these proteins in a peptide-agnostic fashion, both binding sites are predicted with some confidence, with slight preference towards one. When the prediction is done with a native peptide sequence, the confidence in the correct binding site increases. In cases where the correct binding site was initially favoured, the confidence in the alternative binding site also decreases (Supplementary Fig. 11a, b).

To assess the ability of PepNN-Struct to predict multiple binding sites more broadly, we used PepNN-Struct to generate predictions for individual domains and full proteins derived from non-redundant PDB chains that are composed of multiple PRMs. As PepNN-Struct consistently predicts binding sites in individual canonical PRMs (Fig. 4c), correlation of the single domain and full protein predictions indicates that multiple binding sites are predicted. For most examples, the predictions for the single domains and full proteins are well correlated (Supplementary Fig. 11c, d). The one exception is FF domains 4-6 of the TCERG1 protein, where PepNN-Struct only predicts binding sites at FF domains 4 and 5 in the full protein (Supplementary Fig. 11e). Interestingly, previous work has shown that when binding peptides at the C-terminal domain of RNA-polymerase, only residues in domains 4 and 5 contribute to the interaction^42^ (Supplementary Fig. 11f). This supports the notion that PepNN-Struct will preferentially predict sites with a high propensity for peptide binding.

## Discussion

We have developed parallel structure and sequence-based models for the prediction of peptide binding sites. These models, PepNN-Struct and PepNN-Seq, make use of an attention-based deep learning module that is integrated with transfer learning to compensate for the scarcity of peptide-protein complex data. Evaluation on multiple benchmarks shows that PepNN is capable of consistently identifying peptide binding sites. In addition, compared to the leading approach AlphaFold-Multimer, PepNN does not rely on the generation sequence alignments potentially making PepNN more suitable to modelling synthetic interactions. Given that PepNN and AlphaFold often generate successful predictions on different data points, incorporating components from both architectures may furthermore lend itself to the development of a better end-to-end peptide-protein complex modelling approach.

We furthermore demonstrated that PepNN can make accurate peptide-agnostic predictions, and that PepNN outperforms AlphaFold-Multimer in this mode of prediction. This observation is concordant with recent work that has suggested that a protein’s surface contains the majority of information regarding its capacity for biomolecular interactions^41^. Other approaches, trained on negative binding data, are better suited than PepNN to discriminate between identified binding peptides^3,43^. By contrast, PepNN can uniquely be used to score proteins lacking a known peptide ligand to predict their ability to bind peptides. Running this procedure on all proteins in the PDB and the reference human proteome revealed a number of putative novel peptide recognition modules, suggesting that a large portion of the space of PRMs has yet to be characterized. As a demonstration of the model’s capacity to identify novel peptide binders, we showed that residues at a predicted peptide binding site are critical for the interaction between ORF7a and BST-2. The observation that PepNN can make predictions in the absence of a known peptide binder can also be used to discern regions of proteins that can be readily targeted by peptides. PepNN predictions can thus be used to inform the application of high-throughput experimental approaches to different proteins for the purpose of identifying therapeutic peptides.

More generally, the success of PepNN serves as a proof-of-concept of the efficacy of reciprocal attention. This module can effectively be used to model bidirectional relationships between pairs of data points, and can thus be extended to other biomolecular interactions, including protein–protein and protein–DNA interactions. In these cases, sequences or structures of amino acid or nucleic acid sequences can be propagated through sequence or graph attention modules. Reciprocal attention can then be used to effectively relate the residues of the receptor protein to nucleotides or residues in the input, while maintaining symmetry in the updates of the representations. Additionally, the use of transfer learning in the development of PepNN is instructive for the development of approaches to solve related problems. Pre-training on “hot fragment” datasets resulted in large improvements in the performance of PepNN, demonstrating the capacity of deep learning modules to learn transferable biophysical features from these datasets. The generated pre-training datasets of protein fragment-protein complexes are thus a valuable resource for modelling peptide–protein interactions.

## Methods

### Datasets

The training of protein-peptide complexes was generated by filtering complexes in the PDB deposited before 2018/04/30. Crystal structures with a resolution of at least 2.5 Å that contain a chain of more than 30 amino acids in complex with a chain of 25 or fewer amino acids were considered putative peptide-protein complexes. Using FreeSASA^44^, complexes with a buried surface area of less than 400 Å^2^ were filtered out. The sequences of the receptors in the remaining complexes were clustered at a 30% identity threshold using MMseqs2^45^, and the resulting clusters were divided into training and validation sets at proportions of 90% and 10% respectively. To generate the independent test set, the same pipeline was used to process PDB structures with accession dates after 2018/04/30. Test set sequences with more than 30% identity at 70% coverage to a sequence in the training or validation set were removed. The remaining sequences were clustered at 90% identity, and only the centroid of each cluster was kept. The resulting dataset contained 92 sequences.

A similar process was used to generate a dataset of protein fragment-protein complexes. Using the Peptiderive Rosetta protocol^25^, the PDB was scanned for protein fragments of length 5-25 amino acids with a high predicted interface energy when in complex with another chain of at least 50 amino acids. Complexes were filtered out based on the distribution of predicted interface energies from the dataset of real protein-peptide complexes. Only complexes with an interface score less than one standard deviation above the mean of the peptide-protein complex distribution were maintained. The complexes were also filtered by buried surface area. Complexes with less than 400 Å^2^ were once again filtered out. The final dataset contained 406,365 complexes. For data splitting, complexes were again clustered at 30% identity. In both datasets, binding residues were defined as those residues in the protein receptor with a heavy atom within 6 Å from a heavy atom in the interacting chain. Chains with 30% identity at 70% coverage to sequences in the test sets were removed.

In addition to TS092, the models were also tested on benchmark datasets compiled in other studies. This includes the test dataset used to evaluate the Interpep approach^11^ (TS251), the test dataset used to evaluate the PepBind approach^12^ (TS639), and the test dataset used to evaluate SPRINT-Str^26^ (TS125). In these datasets, the input protein was derived from the co-crystallized protein-peptide complex structure. In the case of TS092, an additional benchmark was included with AlphaFold models of the proteins in the complexes.

### Input representation

In the case of PepNN-Struct, input protein structures are encoded using a previously described graph representation^19^, with the exception that additional node features are added to encode the side chain conformation at each residue. In this representation, a local coordinate system is defined at each residue based on the relative position of the Cα to the other backbone atoms^19^. The edges between residues encode information about the distance between the resides, the relative direction from one Cα to another, a quaternion representation of the rotation matrix between the local coordinate systems, and an embedding of the relative positions of the residues in the protein sequence^19^. The nodes include a one-hot representation of the amino acid identity and the torsional backbone angles^19^.

To encode information about the side-chain conformation, the centroid of the heavy side chain atoms at each residue is calculated. The direction of the atom centroid from the Cα is represented using a unit vector based on the defined local coordinate system. The distance is encoded using a radial basis function, similar to the encoding used for inter-residue distances in the aforementioned graph representation^19^. A one-hot encoding is used to represent protein and peptide sequence information. The pre-trained contextualized language model, ProtBert^23^, is used to embed the protein sequence in PepNN-Seq, and this encoding is concatenated to the node features in PepNN-Struct.

### Model architecture

The developed architecture takes inspiration the original Transformer architecture^18^, as well the Structured Transformer, developed for the design of proteins with a designated input structure^19^. Like these models, the PepNN architecture consists of repeating attention and feed forward layers (Fig. 1a). PepNN differs from conventional Transformers, however, in that does not follow an encoder-decoder attention architecture and it makes use of multi-head reciprocal attention. This is an attention-based module that shares some conceptual similarity to a layer that was recently used for salient object detection^46^. Conventional scaled dot attention, mapping queries, represented by matrix $Q$, and key-value pairs, represented by matrices $K$ and $V$, to attention values takes the following form:^18^

1 $${{{\mbox{Attention}}}}\left(Q,\,K,V\right)={{{\mbox{softmax}}}}\left(\frac{Q{K}^{T}}{\sqrt{{d}_{k}}}\right)V$$

In reciprocal attention modules, protein residue embeddings are projected to a query matrix, $Q\in {{\mathbb{R}}}^{{{{{{\rm{n}}}}}}\times {{{{{{\rm{d}}}}}}}_{{{{{{\rm{k}}}}}}}}$ and a value matrix, ${{{{{{\rm{V}}}}}}}_{{{prot}}}\in {{\mathbb{R}}}^{{{{{{\rm{n}}}}}}\times {{{{{{\rm{d}}}}}}}_{{{{{{\rm{v}}}}}}}}$, where $n$ is the number of protein residues. Similarily, the peptide residue embeddings are projected a key matrix, $K\in {{\mathbb{R}}}^{m\times {d}_{k}}$, and a value matrix, ${V}_{{{pep}}}\in {{\mathbb{R}}}^{m\times {d}_{v}}$, where ${m}$ is the number of peptide residues. The resulting attention values are as follows:

2 $${{{\mbox{Attention}}}}_{{{prot}}}\left(Q,\,K,\,{V}_{{{pep}}}\right)={{{\mbox{softmax}}}}\left(\frac{Q{K}^{T}}{\sqrt{{d}_{k}}}\right){V}_{{{pep}}}$$

3 $${{{\mbox{Attention}}}}_{{{pep}}}\left(Q,\,K,\,{V}_{{{prot}}}\right)={{{\mbox{softmax}}}}\left(\frac{K{Q}^{T}}{\sqrt{{d}_{k}}}\right){V}_{prot}$$

In order to extend the definition to multiple heads, the residue encodings are projected multiple times and the resulting attention values are concatenated^18^. The overall model architecture includes alternating self-attention and reciprocal attention layers, with a final set of layers to project the protein residue embedding down to a residue-wise probability score (Fig. 1a). For the purpose of regularization, dropout layers were included after each attention layer.

Model hyperparameters were tuned using random search to optimize the cross-entropy loss on the fragment complex validation dataset. Specifically, eight hyperparameters were tuned; ${d}_{{{{{{\rm{model}}}}}}}$ (the model embedding dimension), ${d}_{i}$ (the dimension of the hidden layer in the feed forward layers), ${d}_{k}$, ${d}_{v}$, the dropout percentage, the number of repetitions of the reciprocal attention module, the number of heads in each attention layer, and the learning rate. In total, 100 random hyperparameter trials were attempted. ${d}_{{{{{{\rm{model}}}}}}}$ was set to 64, ${d}_{i}$ was set to 64, ${d}_{k}$ was set to 64, ${d}_{v}$was set to 128, dropout percentage was set to 0.2, the number of repetitions of the reciprocal attention module was set to 6, and each multi-head attention layer was composed of six heads.

### Training

Training was done using an Adam optimizer with a learning rate of 1e−4 during the pre-training and fine-tuning of PepNN-Struct and a learning rate of 1e−5 when fine-tuning PepNN-Seq. A weighted cross-entropy loss was optimized to take into account the fact that the training dataset is skewed towards non-binding residues. In addition, during fine-tuning, examples were weighted by the inverse of the number of examples in the same sequence cluster. In both the pre-training step with the fragment complex dataset and the training with the peptide complex dataset, early stopping was done based on the validation loss. Training was at most 150,000 iterations during the pre-training step and the at most 35,000 iterations during the fine-tuning step.

### Scoring potential novel peptide binding sites

Peptide-agnostic prediction of proteins in the human proteome and the PDB was performed by providing the model with a protein sequence/structure and a poly-glycine sequence of length 10 as the peptide. The following equation was used to assign scores to putative peptide-binding sites:

4 $$\alpha \frac{1}{n}\mathop{\sum }\limits_{i=1}^{n}{p}_{r}({r}_{i})+\left(1-\alpha \right)p(n/N)$$

where ${p}_{r}({r}_{i})$ is the residue binding probability of the residue with the *i*th highest probability, $N$ is the total number of residues in a protein, $p$ is a pdf of a Gaussian fit to the distribution of binding sites in the training data, and $\alpha$ is a weighting factor. When computing scores using PepNN-Struct, $\alpha$ was set to 0.955. When computing scores using PepNN-Seq $\alpha$ was set to 0.965. Pairwise comparisons were done with the distributions of every PFAM domain to remaining domains with a Wilcoxon rank-sum test and multiple testing correction was done using the Benjamini-Hochberg procedure.

### Identification of peptides that undergo conformational changes

Peptides were aligned to structures in the PDB with the same UniProt identifier as annotated in the Structure Integration with Function, Taxonomy and Sequences resource (SIFTS)^47^. Peptide with a Cα-RMSD of at least 2.5 Å from another structure were annotated as undergoing conformational changes.

### Complex prediction using AlphaFold

To generate protein-peptide complexes with AlphaFold, the sequences were concatenated and 200 was added to the residue index of the peptide residues, as done in ColabFold^32^. Predictions on TS092 were done with templates with dates before 2018/04/30. Predictions on the other datasets, and predictions of receptor structures, were done without templates.

### Statistics and reproducibility

To compare the distribution of scores and metrics across proteins in a dataset, Wilcoxon signed-rank and rank-sum tests were done using the SciPy python library^48^. Multiple testing correction for identifying highly scoring protein domains was done using the statsmodels python package^49^. The DeLong test for identifying differences in ROC curves was done using the pROC R package^50^.

### Protein-protein docking of ORF7a/BST-2

The structure of the SARS-CoV-2 ORF7a encoded accessory protein (PDB ID 6W37) and mouse BST-2/Tetherin Ectodomain (PDB ID 3NI0^51^) were used as input structures for the ClusPro webserver^39,40^. The top ten poses, ranked by population, were used for downstream analysis.

### Cell lines and reagents

HEK293T cells were maintained in DMEM (ATCC) supplemented with 10% FBS and 1% pen/strep/glutamine, and the appropriate selection antibiotics when required. HA antibodies were obtained from Santa Cruz (7392) and Flag antibodies were purchased from Sigma (A8592).

### Western blotting

Transfected cells were scraped from six-well dishes and lysed with lysis buffer (50 mM Tris-HCl pH7.4, 1% Nonidet P-40, 150 mM NaCl, 1 mM EDTA, 1× protease inhibitor mixture (Sigma)) for 30 min at 4 °C. The insoluble pellet was removed following a 10,000 rpm spin for 5 min at 4 °C. Lysates were analyzed by SDS-PAGE/western blot using 4–20% Mini-PROTEAN Tris-glycine gels (Bio-Rad) transferred to PVDF membranes and blocked in 5% milk containing PBS-Tween-20 (0.1%) for 1 h. PVDF membranes were then incubated with specified primary antibodies followed by incubation with horseradish peroxidase-conjugated secondary antibodies (Santa Cruz Biotechnology) and detected using enhanced chemiluminescence (GE Healthcare). HA antibodies were used at a dilution of 1:2000 and Flag antibodies were used at a dilution of 1:1000.

### Flag co-immunoprecipitation

HEK293T cells were cotransfected with Flag-tagged protein and HA-tagged protein. Cells were lysed 48 h after transfections with radioimmune precipitation assay buffer (50 mM Tris-HCl pH7.4, 1% Nonidet P-40, 150 mM NaCl, 1 mM EDTA, 10 mM Na3VO4, 10 mM sodium pyrophosphate, 25 mM NaF, 1× protease inhibitor mixture (Sigma)) for 30 min at 4 °C and coimmunoprecipitated with Flag beads (Clontech). The resulting immunocomplexes were analyzed by Western blot using the appropriate antibodies. Protein samples were separated using 4–20% Mini-PROTEAN Tris-glycine gels (Bio-Rad) transferred to PVDF membranes and blocked in 5% milk containing PBS-Tween-20 (0.1%) for 1 h. PVDF membranes were then incubated with specified primary antibodies followed by incubation with horseradish peroxidase-conjugated secondary antibodies (Santa Cruz Biotechnology) and detected using enhanced chemiluminescence (GE Healthcare).

### Reporting summary

Further information on research design is available in the Nature Research Reporting Summary linked to this article.

## Supplementary information

Supplementary Information

Description of Additional Supplementary Files

Supplementary Data 1

Supplementary Data 2

Supplementary Data 3

Supplementary Data 4

Supplementary Data 5

Supplementary Data 6

Supplementary Data 7

Supplementary Data 8

Supplementary Data 9

Reporting Summary

## Data Availability

Files with PDB codes for the training datasets are included in Supplementary Data 5–7. Full datasets with computed features can be downloaded from http://pepnn.ccbr.proteinsolver.org and can be re-computed using the provided code. Unprocessed blots are provided in Supplementary Fig. 12. Source data for Figs. 2 and 3 are available in Supplementary Data 8 and 9.

## Code availability

The code to run and re-train PepNN is available at https://gitlab.com/oabdin/pepnn^52^.
