# Supplementary material for: PepNN: a deep attention model for the identification of peptide binding sites
Source: Commun Biol. 2022 May 26;5:503. doi: 10.1038/s42003-022-03445-2 (PMC9135736; doi:10.1038/s42003-022-03445-2)
Supplement: Supplementary file 1 — Supplementary Information [file 42003_2022_3445_MOESM1_ESM.pdf]

PepNN: a deep attention model for the identification of peptide binding sites

Supplementary Information

Osama Abdin[1], Satra Nim[2], Han Wen[2], Philip M. Kim[1,2,3,*]

1. Department of Molecular Genetics, University of Toronto, Toronto, ON M5S 3E1, Canada
2. Donnelly Centre for Cellular and Biomolecular Research, University of Toronto, Toronto, ON M5S 3E1, Canada
3. Department of Computer Science, University of Toronto, Toronto, ON M5S 3E1, Canada

* To whom correspondence should be addressed: pi@kimlab.org

Supplementary Tables

Supplementary Table 1: Percentage of data points on which PepNN identifies the ground truth binding site in the top predicted residues

Dataset	Model	%binding sites in top 1 residues	%binding sites in top 5 residues	%binding sites in top 10 residues
TS092	PepNN-Struct	62.0	79.3	82.6
	PepNN-Seq	56.5	80.4	90.2
TS251	PepNN-Struct	53.5	67.1	76.5
	PepNN-Seq	47.4	76.9	86.1
TS639	PepNN-Struct	45.9	65.5	73.4
	PepNN-Seq	30.5	65.2	76.8
TS125	PepNN-Struct	58.4	72.0	79.2
	PepNN-Seq	33.6	66.4	72.8

Supplementary Figures

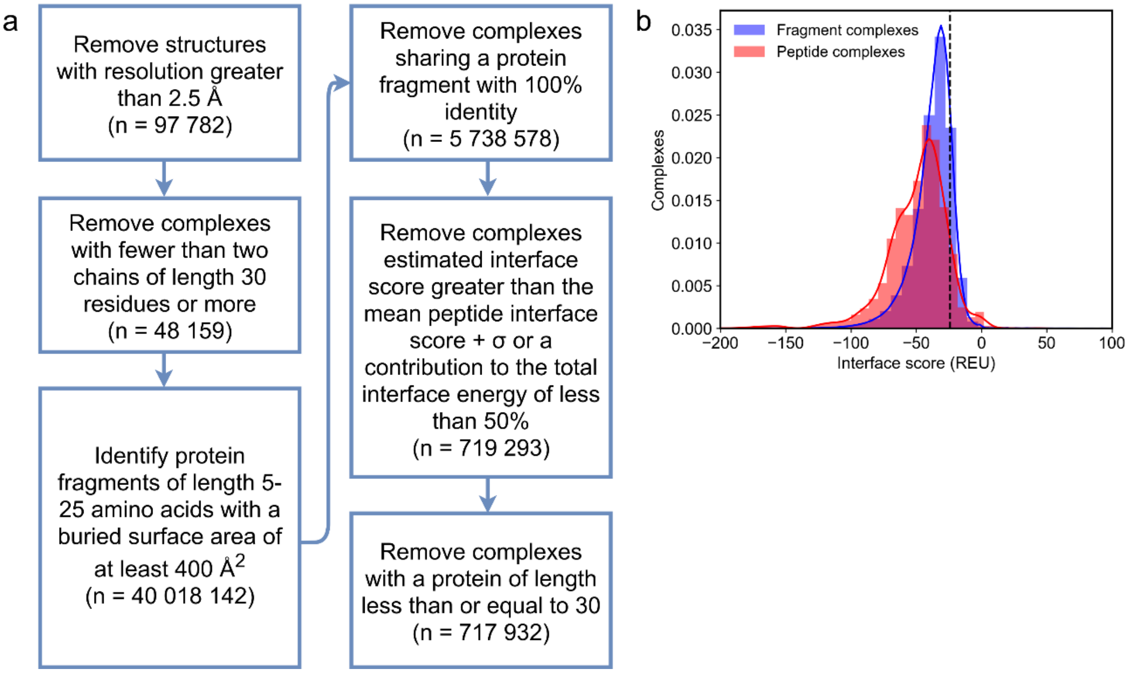

Supplementary Figure 1: Generation and comparison of protein fragment-protein dataset. a. Curation pipeline for generation of a protein fragment-protein dataset. **b.** Comparison of estimated interface distribution of the fragment-protein complexes and the dataset of peptide-protein complexes. The dashed lines indicate the threshold used for filtering fragment-protein complexes.

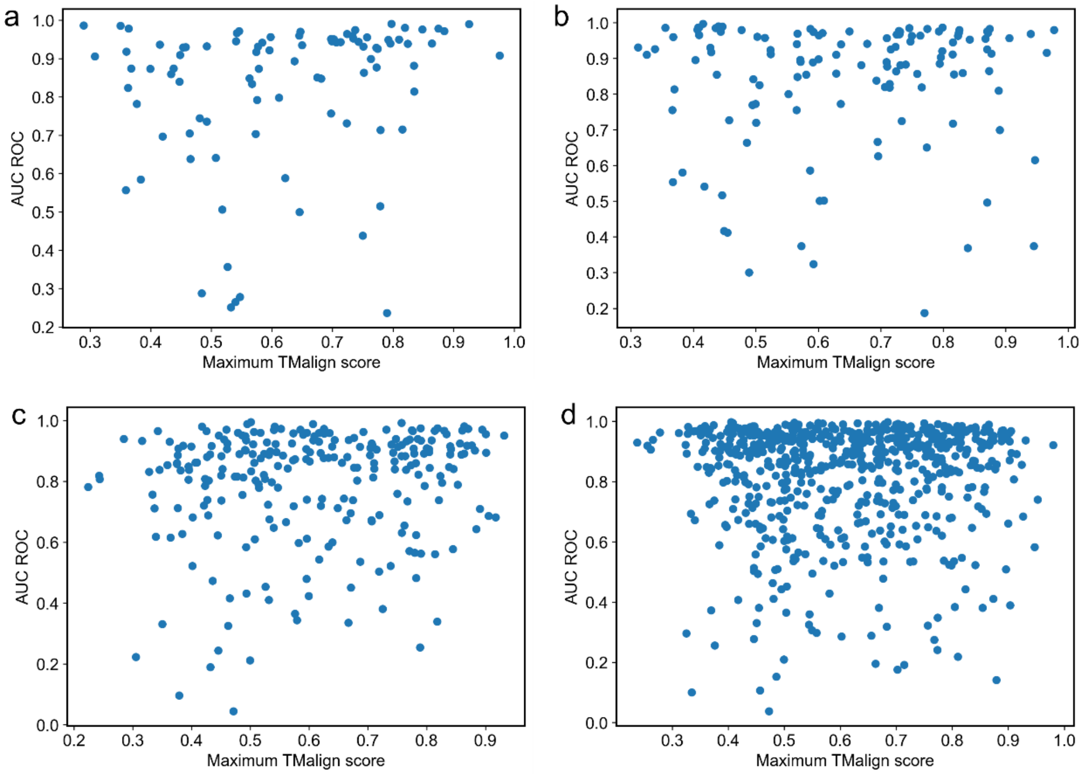

Supplementary Figure 2: Relationship between model performance and structural similarity of test examples to proteins in the pre-training dataset. Relationship between AUC ROC and maximum TMalign score of chains in **a.** TS092 **b.** TS125 **c.** TS251 **d.** TS639 to chains in the pre-training dataset.

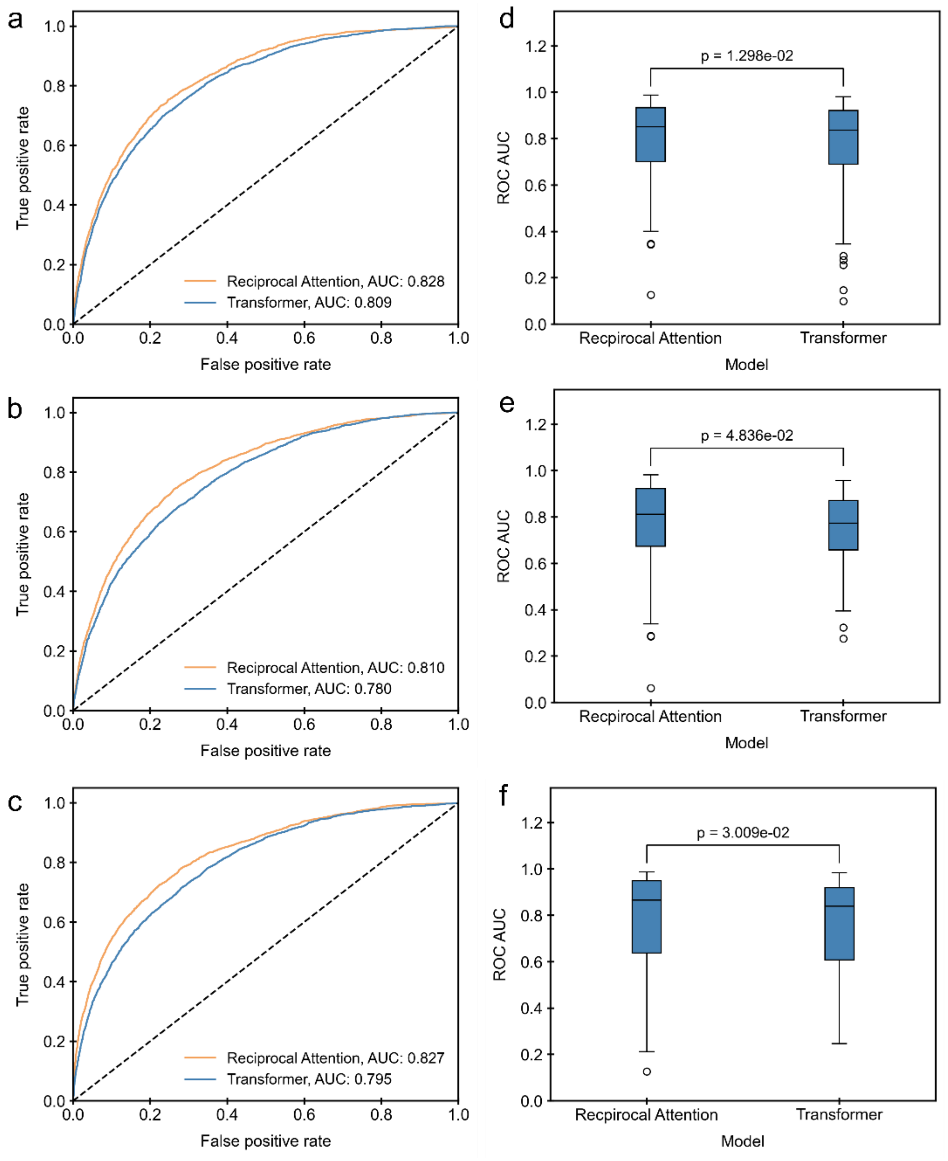

Supplementary Figure 3: Comparison of the performance of PepNN-Struct to a Graph Transformer with the same hyperparameters using difference training procedures. ROC curves on all residues in the dataset **a.** with ProtBert embedding **b.** without ProtBert embeddings **c.** without ProtBert embeddings and pre-trained. Comparison of distribution of ROC AUCs on different input proteins (Wilcoxon signed-rank test) **d.** with ProtBert embedding **e.** without ProtBert embeddings **f.** without ProtBert embeddings and pre-trained. Boxplot centerlines show medians, box limits show upper and lower quartiles, whiskers are 1.5 the interquartile range and points show outliers.

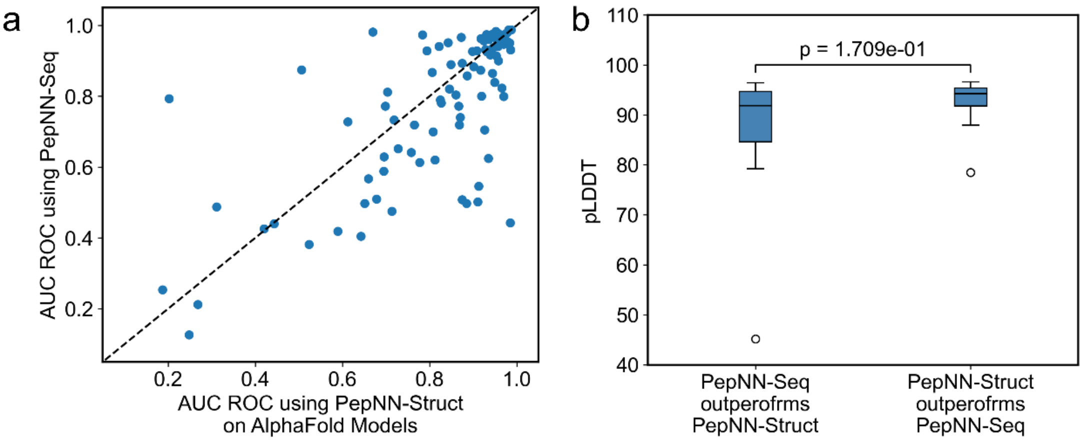

Supplementary Figure 4: Performance of PepNN-Struct on AlphaFold models. a. Comparison of AUC ROC of PepNN-Struct predictions on AlphaFold models and AUC ROC of PepNN-Seq prediction. **b.** pLDDT values of models on which the AUC ROC of PepNN-Seq is at least 0.1 greater than the AUC ROC of PepNN-Struct and vice versa.

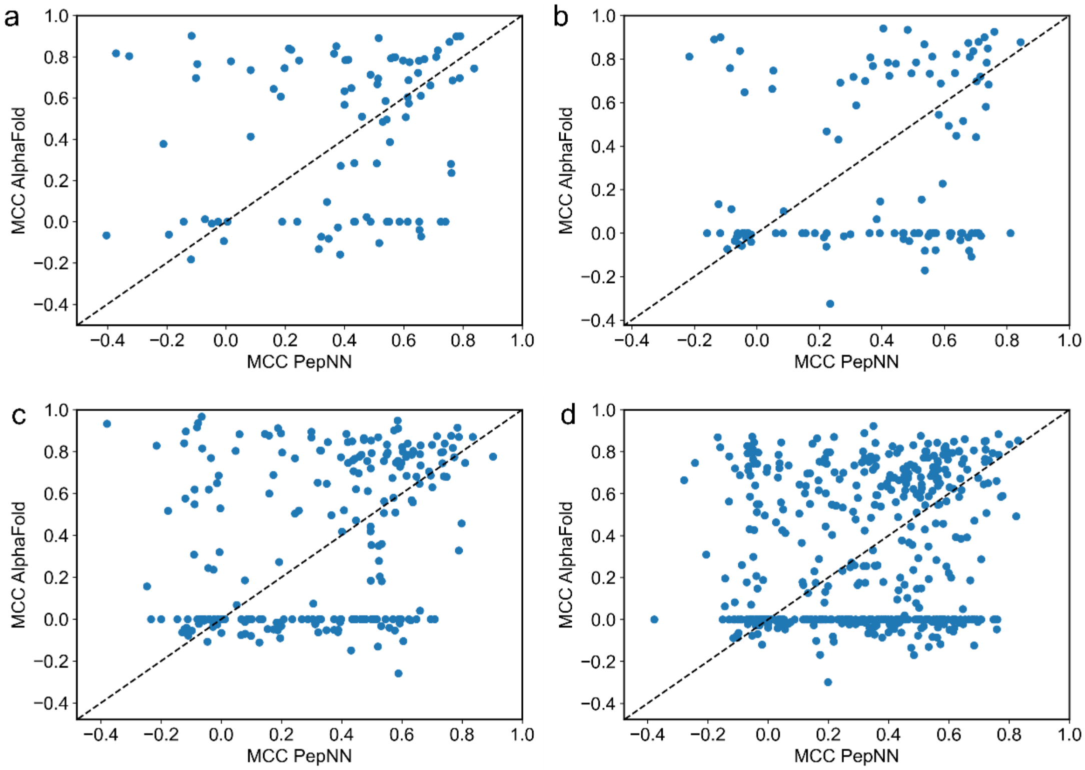

Supplementary Figure 5: Comparison of PepNN and AlphaFold binding site prediction on different dataset. MCC of protein-wise binding site prediction on **a.** TS092 **b.** TS125 **c.** TS251 **d.** TS639

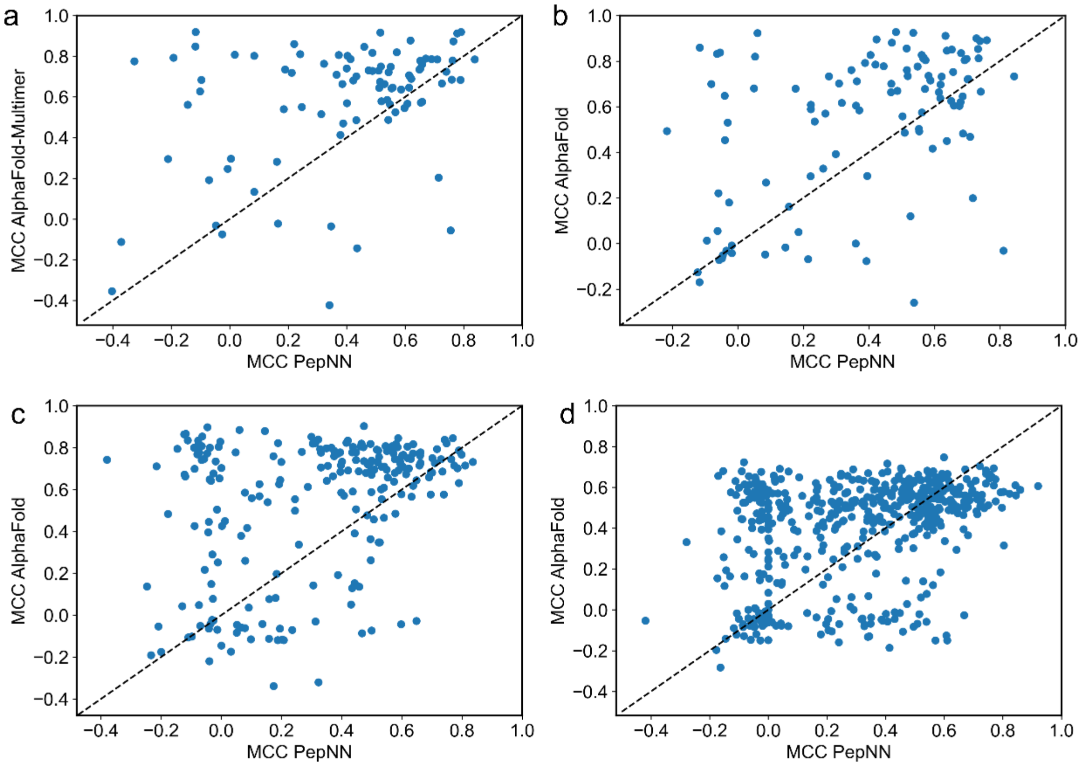

Supplementary Figure 6: Comparison of PepNN and AlphaFold-Multimer binding site prediction on different dataset. MCC of protein-wise binding site prediction on **a.** TS092 **b.** TS125 **c.** TS251 **d.** TS639

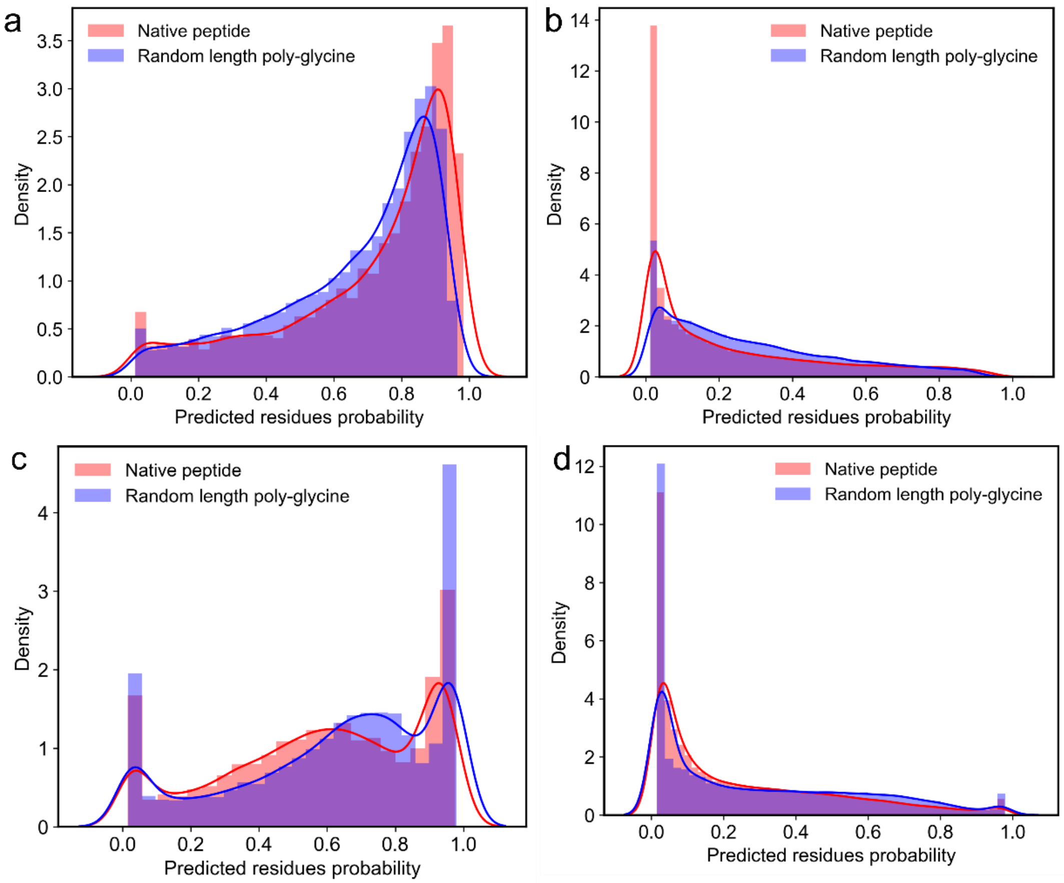

Supplementary Figure 7: Probabilities assigned by PepNN-Struct and PepNN-Seq to different residues with and without the native peptide sequence. a. Probabilities assigned by PepNN-Struct to binding residues. **b.** Probabilities assigned by PepNN-Struct to non-binding residues. **c.** Probabilities assigned by PepNN-Seq to binding residues. **d.** Probabilities assigned by PepNN-Seq to non-binding residues.

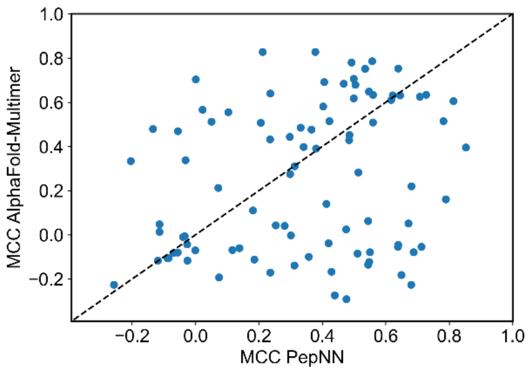

Supplementary Figure 8: Comparison of PepNN and AlphaFold-Multimer binding site prediction with poly-glycine inputs on TS092.

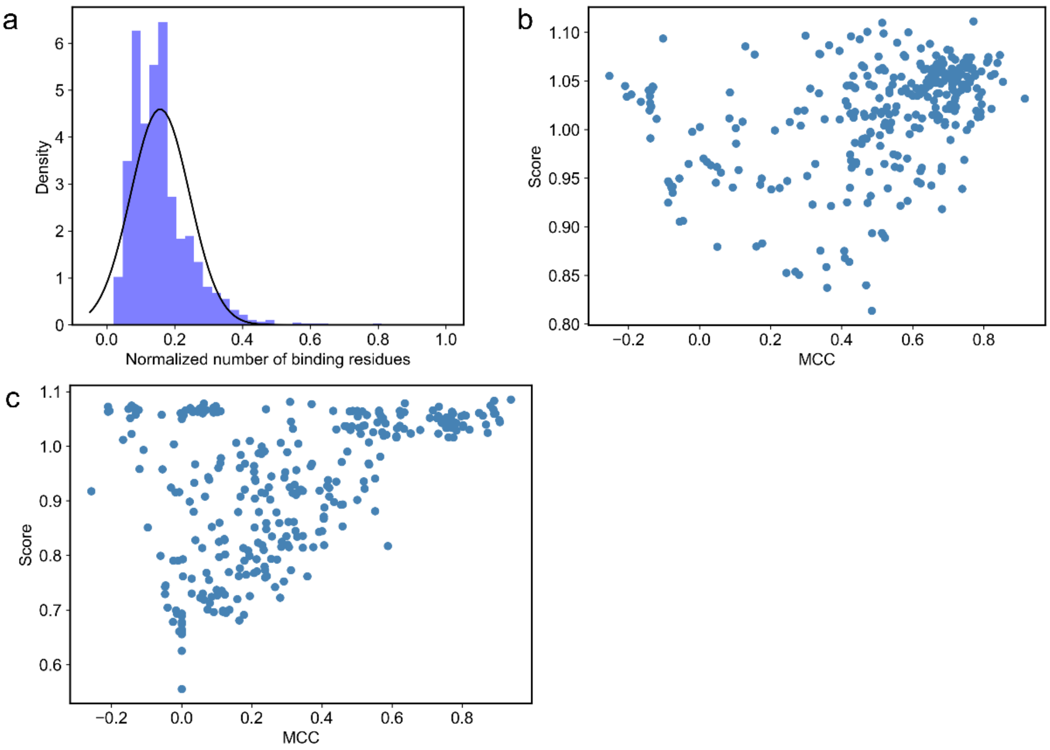

Supplementary Figure 9: Scoring of domains for peptide binding using PepNN. a. The percentage of binding residues in different examples in the training dataset. **b.** Relationship between scores assigned by PepNN-Struct and MCC of predictions. **c.** Relationship between scores assigned by PepNN-Seq and MCC of predictions.

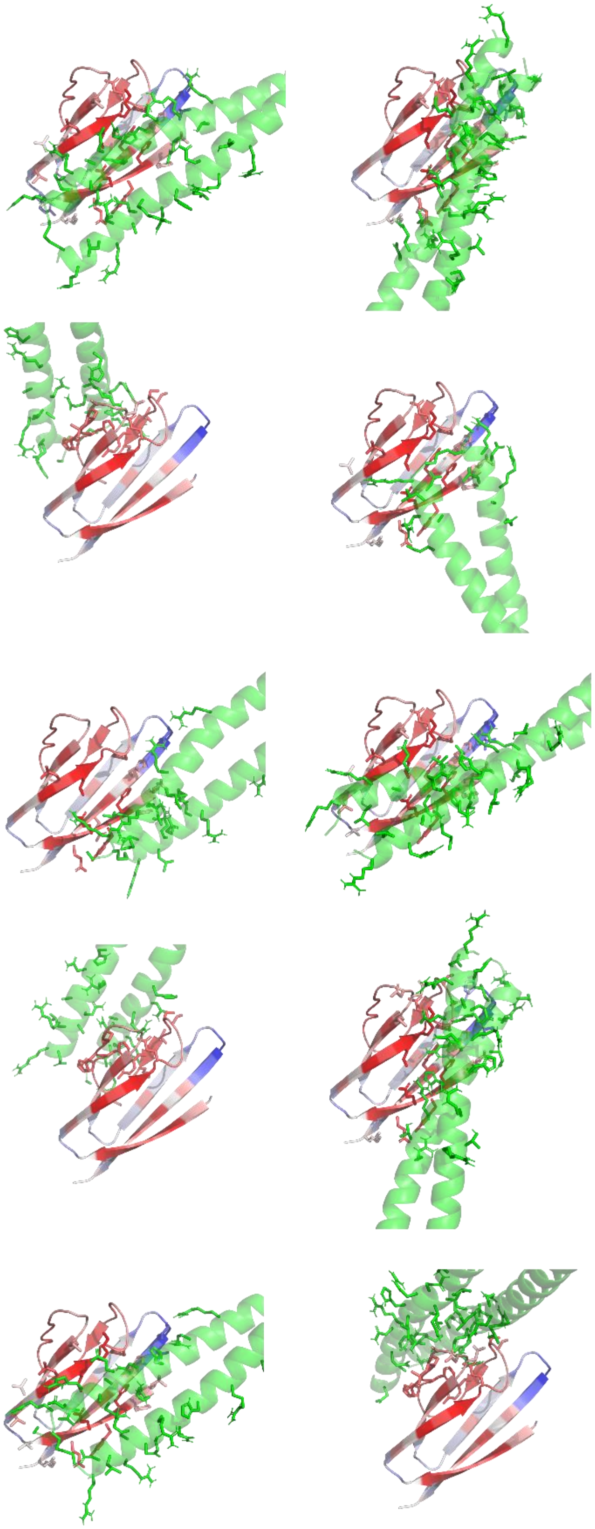

Supplementary Figure 10: Complex conformations generated by docking BST-2 against ORF7a.

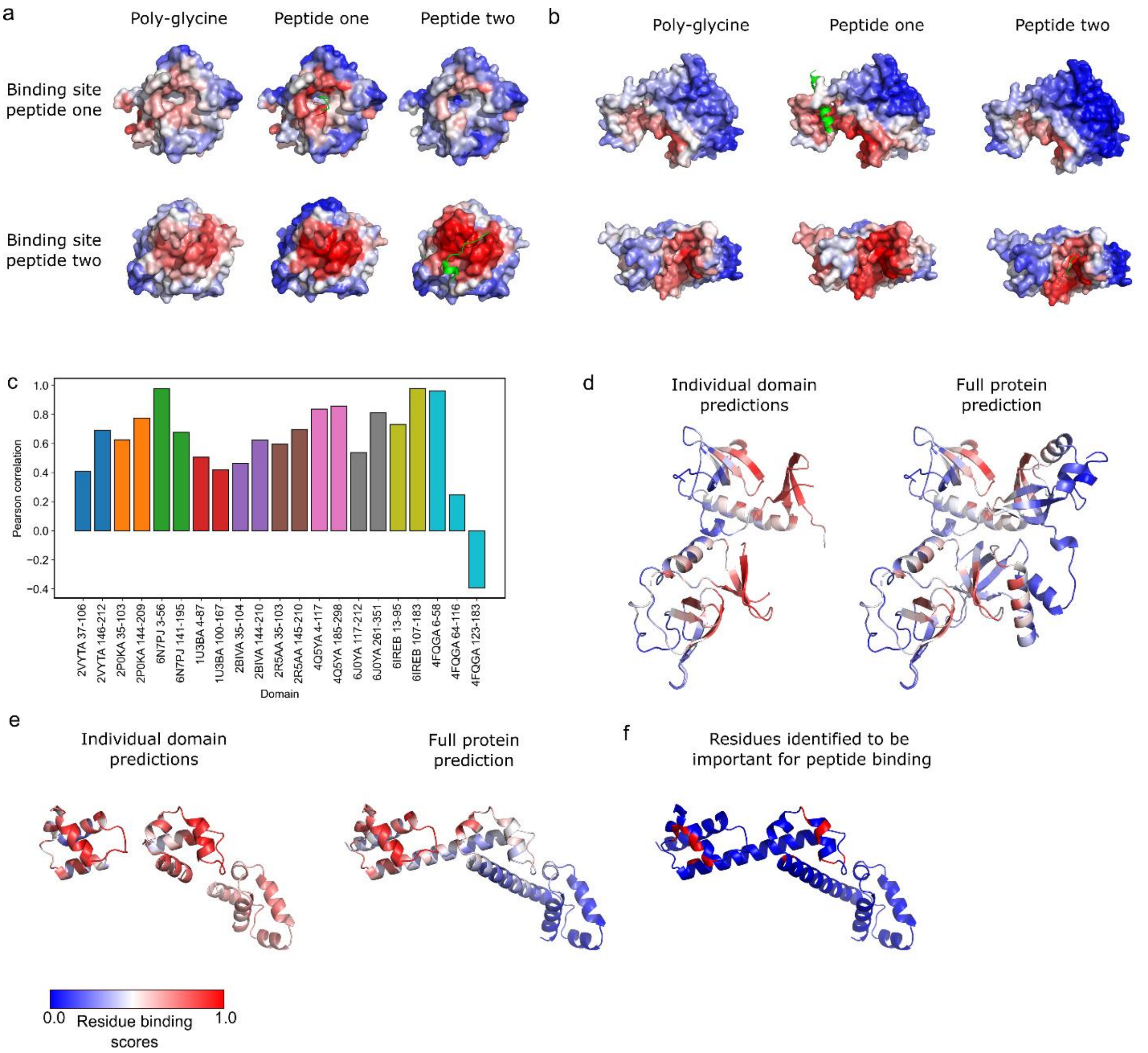

Supplementary Figure 11: Multiple peptide binding site prediction using PepNN-Struct. a. Binding site predictions on protein X-ray repair cross-complementing protein 5 (PDB code 6TYT) using different input peptides. **b.** Binding site predictions on Small vasohibin-binding protein (PDB code 6J8F) using different input peptides **c.** Pearson correlation between residue-wise predictions on individual PRMs and full proteins containing multiple PRMs. **d.** Predictions on individual Tudor domains of the Maternal protein tudor protein and the full chain (PDB Code 4Q5Y). **e.** Predictions on individual FF domains of the Transcription elongation regulator 1 protein and the full chain (PDB Code 4FQG). **f.** Residues identified as important for peptide binding by Liu et al. 2013[7].

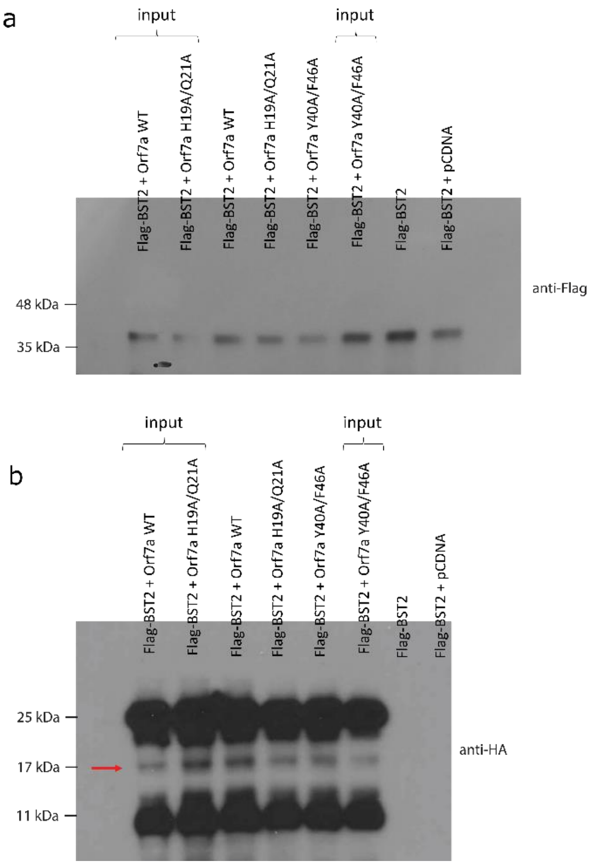

Supplementary Figure 12: Unprocessed blots for co-immunoprecipitation of wild type and mutant ORF7A with BST-2. a. Anti-Flag blot. **b.** Anti-HA blot.

Supplementary References

1. Williams, W. V., Kieber-Emmons, T., VonFeldt, J., Greene, M. I. & Weiner, D. B. Design of bioactive peptides based on antibody hypervariable region structures: Development of conformationally constrained and dimeric peptides with enhanced affinity. *J. Biol. Chem.* **266**, (1991).
2. Williams, W. V *et al.* Sequences of the cell-attachment sites of reovirus type 3 and its anti-idiotypic/antireceptor antibody: modeling of their three-dimensional structures. *Proc. Natl. Acad. Sci.* **85**, 6488–6492 (1988).
3. Taub, R. *et al.* A monoclonal antibody against the platelet fibrinogen receptor contains a sequence that mimics a receptor recognition domain in fibrinogen. *J. Biol. Chem.* **264**, (1989).
4. Dunbar, J. *et al.* SAbDab: the structural antibody database. *Nucleic Acids Res.* **42**, D1140–D1146 (2014).
5. Adolf-Bryfogle, J., Xu, Q., North, B., Lehmann, A. & Dunbrack Jr, R. L. PyIgClassify: a database of antibody CDR structural classifications. *Nucleic Acids Res.* **43**, D432–D438 (2015).
6. McGibbon, R. T. *et al.* MDTraj: A Modern Open Library for the Analysis of Molecular Dynamics Trajectories. *Biophys. J.* **109**, 1528–1532 (2015).
7. Liu, J., Fan, S., Lee, C.-J., Greenleaf, A. L. & Zhou, P. Specific interaction of the transcription elongation regulator TCERG1 with RNA polymerase II requires simultaneous phosphorylation at Ser2, Ser5, and Ser7 within the carboxyl-terminal domain repeat. *J. Biol. Chem.* **288**, 10890–10901 (2013).
