# Supplementary material for: PepNN: a deep attention model for the identification of peptide binding sites
Source: Commun Biol. 2022 May 26;5:503. doi: 10.1038/s42003-022-03445-2 (PMC9135736; doi:10.1038/s42003-022-03445-2)
Supplement: Supplementary file 2 — Description of Additional Supplementary Files [file 42003_2022_3445_MOESM2_ESM.pdf]

Description of Additional Supplementary Files

File name: Supplementary Data 1
Description: Scores assigned to different domains in the PDB by PepNN-Struct

File name: Supplementary Data 2
Description: Scores assigned to different domains in the reference human proteome by PepNN-Seq

File name: Supplementary Data 3
Description: Domains assigned scores higher or lower than the overall distribution by PepNN-Struct

File name: Supplementary Data 4
Description: Domains assigned scores higher or lower than the overall distribution by PepNN-Seq

File name: Supplementary Data 5
Description: Fragment complex dataset

File name: Supplementary Data 6
Description: Peptide complex dataset

File name: Supplementary Data 7
Description: Test peptide complexes

File name: Supplementary Data 8
Description: The source data for Figure 2

File name: Supplementary Data 9
Description: The source data for Figure 3
