# Supplementary material for: PepNN: a deep attention model for the identification of peptide binding sites
Source: Commun Biol. 2022 May 26;5:503. doi: 10.1038/s42003-022-03445-2 (PMC9135736; doi:10.1038/s42003-022-03445-2)
Supplement: Supplementary file 12 — Reporting Summary [file 42003_2022_3445_MOESM12_ESM.pdf]

nature portfolio

Corresponding author(s): Philip M Kim

Last updated by author(s): Apr 19, 2022

Reporting Summary

Nature Portfolio wishes to improve the reproducibility of the work that we publish. This form provides structure for consistency and transparency in reporting. For further information on Nature Portfolio policies, see our Editorial Policies and the Editorial Policy Checklist.

Statistics

For all statistical analyses, confirm that the following items are present in the figure legend, table legend, main text, or Methods section.

n/a	Confirmed	
☐	☒	The exact sample size (n) for each experimental group/condition, given as a discrete number and unit of measurement
☐	☒	A statement on whether measurements were taken from distinct samples or whether the same sample was measured repeatedly
☐	☒	The statistical test(s) used AND whether they are one- or two-sided<br>*Only common tests should be described solely by name; describe more complex techniques in the Methods section.*
☐	☒	A description of all covariates tested
☐	☒	A description of any assumptions or corrections, such as tests of normality and adjustment for multiple comparisons
☐	☒	A full description of the statistical parameters including central tendency (e.g. means) or other basic estimates (e.g. regression coefficient) AND variation (e.g. standard deviation) or associated estimates of uncertainty (e.g. confidence intervals)
☐	☒	For null hypothesis testing, the test statistic (e.g. F, t, r) with confidence intervals, effect sizes, degrees of freedom and P value noted<br>*Give P values as exact values whenever suitable.*
☒	☐	For Bayesian analysis, information on the choice of priors and Markov chain Monte Carlo settings
☒	☐	For hierarchical and complex designs, identification of the appropriate level for tests and full reporting of outcomes
☐	☒	Estimates of effect sizes (e.g. Cohen's d, Pearson's r), indicating how they were calculated

Our web collection on statistics for biologists contains articles on many of the points above.

Software and code

Policy information about availability of computer code

Data collection	Custom software used to collect and analyze the data is available at https://gitlab.com/oabdin/pepnn.
Data analysis	Custom software used to collect and analyze the data is available at https://gitlab.com/oabdin/pepnn.

For manuscripts utilizing custom algorithms or software that are central to the research but not yet described in published literature, software must be made available to editors and reviewers. We strongly encourage code deposition in a community repository (e.g. GitHub). See the Nature Portfolio guidelines for submitting code & software for further information.

Data

Policy information about availability of data

All manuscripts must include a data availability statement. This statement should provide the following information, where applicable:

- Accession codes, unique identifiers, or web links for publicly available datasets
- A description of any restrictions on data availability
- For clinical datasets or third party data, please ensure that the statement adheres to our policy

Files with PDB codes for the training datasets are included in Supplementary Data 5-7. Full datasets with computed features can be downloaded from http://pepnn.ccbr.proteinsolver.org and can be re-computed using the provided code. Unprocessed blots are provided in Supplementary Figure 12.

Field-specific reporting

Please select the one below that is the best fit for your research. If you are not sure, read the appropriate sections before making your selection.

☒ Life sciences ☐ Behavioural & social sciences ☐ Ecological, evolutionary & environmental sciences

For a reference copy of the document with all sections, see nature.com/documents/nr-reporting-summary-flat.pdf

Life sciences study design

All studies must disclose on these points even when the disclosure is negative.

Sample size	The sample sizes used in the computational study were based on the availability of protein-peptide complex information in the PDB. Co-immunoprecipitation and western blotting was performed twice.
Data exclusions	Redundant samples were removed to ensure no overlap between train, validation and test sets.
Replication	The results of the computational study can be reproduced using the provided data and code. Co-immunoprecipitation and western blotting was replicated.
Randomization	Available data was clustered by similarity and clusters were split randomly between train and validation. Recent structures (available after 2018) were used as test data.
Blinding	N/A

Reporting for specific materials, systems and methods

We require information from authors about some types of materials, experimental systems and methods used in many studies. Here, indicate whether each material, system or method listed is relevant to your study. If you are not sure if a list item applies to your research, read the appropriate section before selecting a response.

Materials & experimental systems

n/a	Involved in the study	
☐	☒	Antibodies
☐	☒	Eukaryotic cell lines
☒	☐	Palaeontology and archaeology
☒	☐	Animals and other organisms
☒	☐	Human research participants
☒	☐	Clinical data
☒	☐	Dual use research of concern

Methods

n/a	Involved in the study	
☒	☐	ChIP-seq
☒	☐	Flow cytometry
☒	☐	MRI-based neuroimaging

Antibodies

Antibodies used	HA antibodies were obtained from Santa Cruz (7392) (dilution 1:2000) and Flag antibodies were purchased from Sigma (A8592) (dilution (1:1000).
Validation	HA and Flag antibodies were used for immunoprecipitation experiments. The resulting immunocomplexes were analyzed by Western blot using the appropriate antibodies. Control experiments with only HA and Flag tag constructs were used to determine the specificity of the antibodies.

Eukaryotic cell lines

Policy information about cell lines

Cell line source(s)	HEK293T cells were obtained from ATCC.
Authentication	none of the cell lines were authenticated.
Mycoplasma contamination	All cell lines tested negative for mycoplasma contamination.
Commonly misidentified lines (See ICLAC register)	N/A
